# Chronic *Schistosoma japonicum* Infection Reduces Immune Response to Vaccine against Hepatitis B in Mice

**DOI:** 10.1371/journal.pone.0051512

**Published:** 2012-12-14

**Authors:** Lin Chen, Wen-qi Liu, Jia-hui Lei, Fei Guan, Man-jun Li, Wen-jian Song, Yong-long Li, Ting Wang

**Affiliations:** 1 Department of Parasitology, Tongji Medical College, Huazhong University of Science and Technology, Wuhan, China; 2 Wuhan Institutes of Biomedical Sciences, Jianghan University, Wuhan, China; 3 Department of Parasitology, Jianghan University, Wuhan, China; Queensland Institute of Medical Research, Australia

## Abstract

**Background:**

Hepatitis B and schistosomiasis are most prevalent in Africa and Asia, and co-infections of both are frequent in these areas. The immunomodulation reported to be induced by schistosome infections might restrict immune control of hepatitis B virus (HBV) leading to more severe viral infection. Vaccination is the most effective measure to control and prevent HBV infection, but there is evidence for a reduced immune response to the vaccine in patients with chronic schistosomiasis japonica.

**Methodology/Principal Findings:**

In this paper, we demonstrate in a mouse model that a chronic *Schistosoma japonicum* infection can inhibit the immune response to hepatitis B vaccine (HBV vaccine) and lead to lower production of anti-HBs antibodies, interferon-γ (IFN-γ) and interleukin-2 (IL-2). After deworming with Praziquantel (PZQ), the level of anti-HBs antibodies gradually increased and the Th2-biased profile slowly tapered. At 16 weeks after deworming, the levels of anti-HBs antibodies and Th1/Th2 cytokines returned to the normal levels.

**Conclusions/Significance:**

The results suggest that the preexisting Th2-dominated immune profile in the host infected with the parasite may down–regulate levels of anti-HBs antibodies and Th1 cytokines. To improve the efficacy of HBV vaccination in schistosome infected humans it may be valuable to treat them with praziquantel (PZQ) some time prior to HBV vaccination.

## Introduction

Poor immune responses after vaccination have been reported for both viral and bacterial vaccines [1]–[4]. Besides genetic predisposition, immunosuppression, and certain chronic illnesses [5], helminthic infections may be a contributing cause for absent or weak responsiveness to the vaccines [6].

Hepatitis B is widespread in the world, especially in central Asia, Southeast Asia, sub-Saharan Africa, and the Amazon Basin. Globally, at least 2 billion people have experienced an infection with the HBV, about 380 million people are chronic carriers, and approximately 620,000 people die each year from acute and chronic sequelae secondary to HBV infection [7], [8]. Vaccination is the measure that is most effective in reducing the incidence of hepatitis B [9]. Antibodies against the hepatitis B surface antigen (anti-HBs) induced by a HBV vaccine might mediate important antiviral effector functions because anti-HBs are virus neutralizing [10], [11]. Although vaccination against HBV is highly successful, 5% to 10% of individuals do not experience a response with an adequate level of anti-HBs [5]. Recent studies showed that helminthic infections could impair the immune response of the host to TB and HIV [12]. Epidemiological investigations in China found that the rates of absent or weak responses to the HBV vaccine are higher in rural than that in urban children (60.1% v.s 5–10%) [13], [14]. The results suggested that the failure may be related to parasitic infections.

Schistosomiasis is also widespread in tropic and sub-tropic areas. According to World Health Organization estimates, 779 million people are at risk of schistosomiasis, and 207 million people are infected in 76 countries [15], [16], [17]. Effects of schistosomal infections on vaccination efficacy have been reported. Sabin and colleagues [18] found that tetanus toxoid (TT)-specific Th1-like responses were low in schistosome-infected subjects in comparison to non-infected controls. Van Riet et al. [19] found that children with concurrent schistosomiasis showed reduced IFN-*γ* responses to TT compared to non-infected subjects after tetanus vaccination. In addition, these children received an influenza vaccine and similarly it was found that the IFN-*γ* response to influenza was higher in non-infected children, whereas IL-5 and IL-13 production was increased in infected children. In China 62.4% of patients with chronic or advanced schistosomiasis are infected with HBV [20]. In a previous study we found absent or weak responses to a HBV vaccine under a standard three-dose immunization schedule in 83% (20/24) of patients with a chronic *S. japonicum* infection, whereas the corresponding value was 7.7% for healthy persons [21]. We hypothesized that the absent or weak-responses to the HBV vaccine could be related to the schistosomal infection.

In the present paper, we studied the effects of *S. japonicum* infection and termination of the infection with PZQ on the protective efficacy of hepatitis B vaccine.

## Materials and Methods

### Ethics Statement

All animal work was approved by the Hubei Provincial Department of Science and Technology (ID SCXK 2008-0003) and the Animal Care Committee of the Tongji Medical College (ID 2009-S226), and it complied with the guidelines of the Animal Care Committee, Chinese Academy of Sciences (Animal Welfare Assurance #A5748-01). All the operated mice were performed under anesthesia.

### Mice and Parasites

Male BALB/c mice, 6–8 weeks of age, were purchased from the Wuhan Institute of Biologic Products (Wuhan, China). The life cycle of a Chinese strain of *S. japonicum* was maintained in a laboratory of the Hunan Institute of Schistosomiasis Control. Cercariae of *S. japonicum* were shed from *Oncomelania hupensis* snails.

### *S. japonicum* Infection

Mice were randomly divided into 7 groups (each group with 10 mice): control group, acute infection group (2 weeks after infection), chronic infection group (8 weeks after infection), PZQ4W group (4 weeks after treatment with PZQ), PZQ8W group (8 weeks after treatment), PZQ12W group (12 weeks after treatment) and PZQ16W group (16 weeks after treatment).

All mice were percutaneously infected under anesthesia with 25 cercariae of *S. japonicum* placed on the shaved abdominal skin. Mice in the control group remained un-infected.

**Figure 1:**
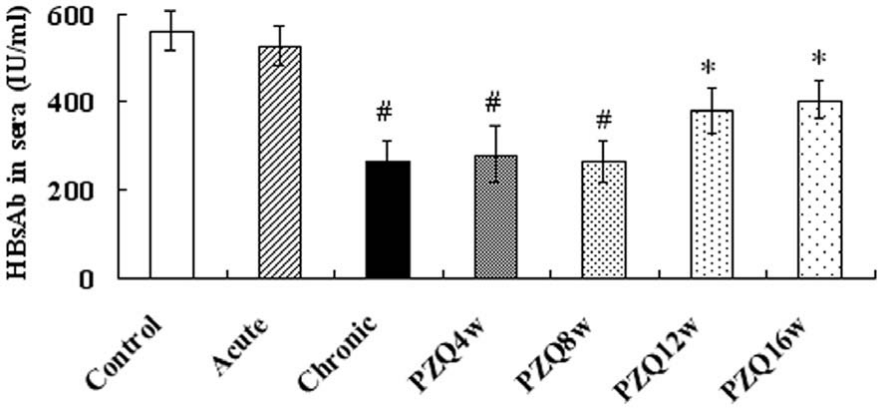
Levels of anti-HBsAg antibody in sera of mice. Anti-HBsAg antibody was individually determined by a commercial enzyme-linked immunoassay. Two separate experiments (n = 10 mice/group) were performed and the data from two independent experiments were pooled and so each value shown represents the means and SE of 20 value. * P<0.05 versus control group. ^#^ P<0.01 versus control group.

### Immunization Scheme

All animals were vaccinated with a recombinant yeast derived HBV vaccine (Kangtai Biologic Products LTD, China), 0.1 µg/g body weight, via dorsal subcutaneous injection, for three times at intervals of 14 days. The acute and chronic infection groups were vaccinated at 2 and 8 weeks, respectively, after infection. Mice of groups PZQ 4 w, PZQ 8 w, PZQ 12 w and PZQ 16 w were given the first vaccination at 4, 8, 12 and 16 weeks, respectively, after drug treatment.

### Drug Treatment

All mice in the PZQ groups were treated at 8 weeks after infection. PZQ (Bayer, Leverkusen, Germany) was suspended in 2% aqueous solution of Cremophor (Sigma, St. Louis, USA) and administered intragastrically on two consecutive days with a total dose of 500 mg/kg body weight [20].

### Serum Samples and Spleen Cells

Two weeks after the final vaccination, mice were lethally anaesthetized by peritoneal injection of 0.67% sodium pentobarbital in physiological saline (10 µl per gram of body weight). Blood was taken from the orbital veins, the sera were collected and stored at −20°C for measurement of Anti-HBsAg antibody and cytokines. Spleens were removed and individually divided to prepare single-cell suspensions from individual mice [22] as well as tissue for RNA isolation (see below).

### Assay for Anti-HBsAg Antibody in Serum

Anti-HBsAg antibody were determined in the individual serum samples (1∶10 diluted with PBS) by a commercial enzyme-linked immunoassay (Shanghai kehua bio-engineering LTD, China) according to the manufacturer’s instructions. Optical density (OD) values were determined at 450 nm in an ELISA reader (RT-6000, Rayto, Shenzhen, China).

**Figure 2:**
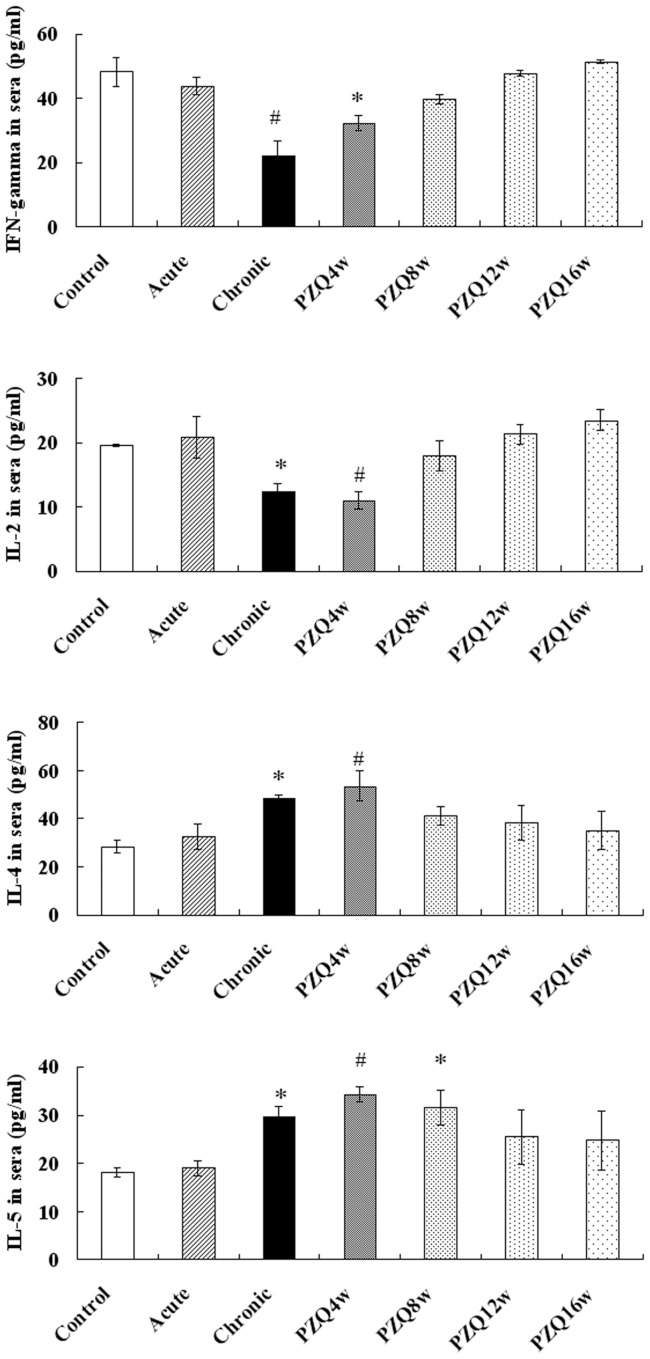
Th1 and Th2 cytokines in sera of mice. The levels of IFN-γ IL-2, IL-4, and IL-5 were determined in individual sera (n = 10 mice/group) by commercially available ELISA kits. The experiment was performed twice and each value shown represents the mean and SE of 20 value. * P<0.05 versus control group. ^#^ P<0.01 versus control group.

### Measurement of Cytokine Production in Serum

The levels of IFN-γ, IL-12, IL-4, IL-5 and IL-10 in individual sera were determined using commercially available ELISA kits (eBioscience, USA). The reactions were measured at 450 nm in the ELISA reader. The concentrations of cytokines in samples were calculated by standard curves constructed with known amounts of mouse recombinant IFN-γ, IL -2, IL-4 and IL-5 (eBioscience, USA) and results are expressed in pictograms per ml.

### Measurement of Cytokine Production in Spleen Cell Culture Supernatants

Splenocytes (2×10^5^ cells/well) were cultured in triplicate wells in 96-well plates, using RPMI-1640 medium containing 5% FCS at 37°C under 5% CO_2_, and incubated in the absence or presence of 2 µg/ml of purified recombinant HBsAg without vaccine additives (Kangtai Biologic Products LTD, China) plus 5 µg/ml of ConA (Sigma, USA) for 72 h. The supernatants were collected and the concentrations of IFN-γ, IL-2, IL-4 and IL-5 were determined with the respective ELISA Kits and standard curves (see above).

**Figure 3:**
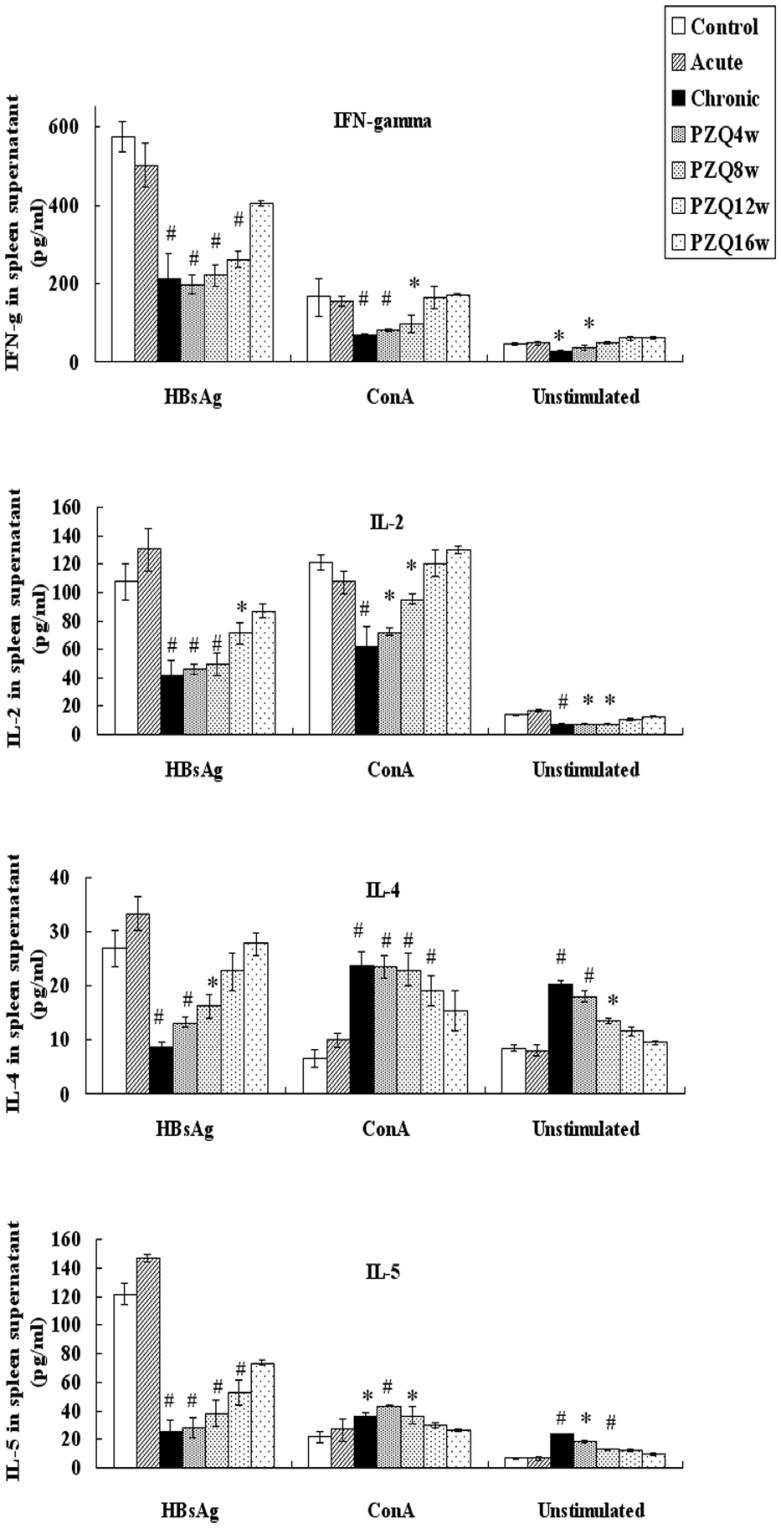
Concentrations of Th1and Th2 cytokines in spleen cell culture supernatants of mice. Spleen cells derived from the different groups at different times were tested under the same conditions. The experiment was performed twice (n = 10 mice/group) and each value shown represents the mean and SE of 20 value. P<0.05 versus control group. ^#^ P<0.01 versus control group.

**Figure 4:**
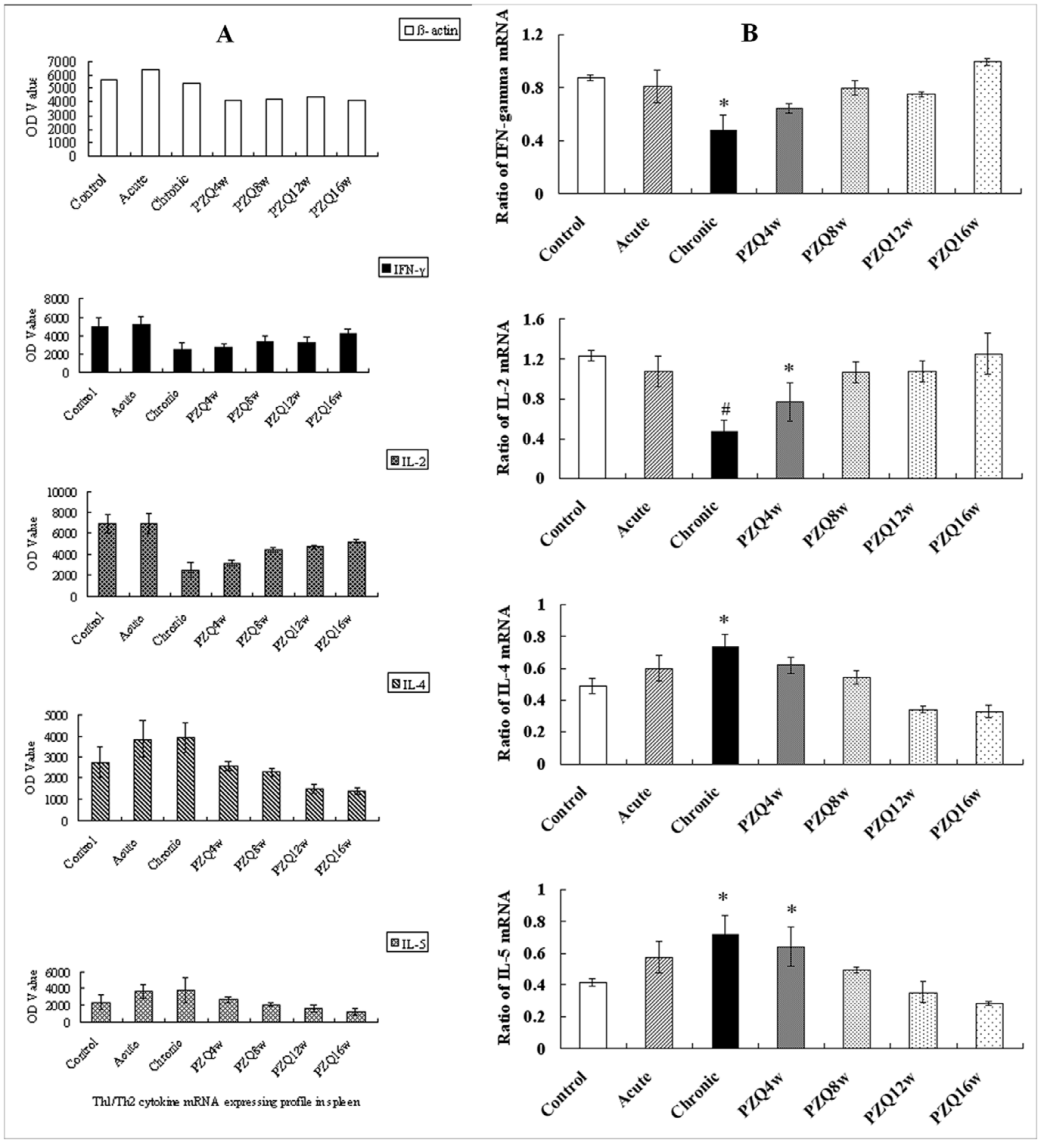
Levels of Th1/Th2 cytokine mRNA RT-PCR production of mice spleens. The PCR amplified fragments were analyzed on agarose gel. The optical density of each band was measured with a computer-assisted image analysis system. Relative expressions of Th1/Th2 cytokine mRNA were analyzed by the ratio of the band density of its amplified fragment over that of β-actin. A: OD values of RT-PCR products from Th1/Th2 cytokine mRNA and β-actin. B: The ratios of Th1/Th2 cytokine mRNA expression. The data in Fig. 4B does not necessarily reflect the exact ratios of each cytokine mRNA to b-actin, because PCR amplification is not exponential at high cycle numbers. *P<0.05 versus control group, ^#^ P<0.01 versus control group.

### RT-PCR Procedures for Cytokines mRNA

Total RNA was isolated from individual spleen tissues using RNArose reagent (Watsonbiot, Shanghai, China) and reverse-transcribed into cDNA using ReverTra Ace-α-TM (Toyobo, Osaka, Japan). All primers were designed by Primer 5.0 and synthesized by Invitrogen Biotech, Shanghai, China. The primers were:

IFN-γ (product size: 421 bp), forward primer (5′ TTGGA CCCTC TGACT T 3′), reverse primer (5′ CAAAC TTGGC AATAC TC 3′);

IL-2 (product size: 361 bp), forward primer (5′ TACAG CGGAA GCACA GCA 3′), reverse primer (5′ CCTTA GAAAG TCCAC CACA 3′);

IL-4 (product size: 380 bp), forward primer (5′ GTTGT CATCC TGCTC TTC 3′), reverse primer (5′ ATGCT CTTTA GGCTT TCC 3′);

IL-5 (product size: 591 bp), forward primer (5′ AGGCT TCCTG TCCCT ACT 3′), reverse primer (5′ CCATC TCCAG CACTT CAT 3′).

The housekeeping gene β-actin (product size: 542 bp) with the forward primer (5′ ATGGG TCAGA AGGAC TCCTA TG 3′) and the reverse primer (5′ ATCTC CTGCT CGAAGT CTAGA G 3′) was used as a control.

PCR was performed in a 25 µl reaction volume using 2× Taq PCR Mastermix (Tiangen Biotech, Beijing, China). The cycle parameters were as follows: pre-heating at 95°C for 5 minutes, denaturation at 94°C for 50 seconds, annealing at 49°C for 45 seconds and extension at 72°C for 1 minute. A total of 40 cycles were run, and each program was ended with 10 minutes at 72°C. The final PCR products were analyzed by agarose gel electrophoresis. The optical density of each band was measured with a computer-assisted image analysis system 212 style (Carestream Health, Canada).

### Statistical Analysis

All data are expressed as mean ± SE and were analyzed by one-way ANOVA analysis. Turkey’s post-hoc test was carried out to determine, whether P values between two groups were significantly different. P<0.05 was considered significant.

## Results

### Anti-HBsAg Antibody Levels in Serum

Two weeks after the final vaccination, the levels of specific anti-HBsAg antibody were determined in the serum samples. The results show that the mean concentration of anti-HBsAg antibody in the mice with a chronic infection group was significantly lower than that in the un-infected control and acute infection group (P<0.05), and there was no significant difference between the acute infection and control groups (P>0.05). After treatment with PZQ, the means of anti-HBsAg antibody levels slowly rose. At 12 w and 16 w after treatment, the anti-HBsAg antibody reached the level of the control group (P>0.05) (Fig. 1).

### Th1/Th2 Cytokine Profile in Serum

As shown in Fig. 2, the concentrations of Th1 cytokines (IFN-γ and IL-2) in sera of the chronic infection groups were significantly lower than those in all other groups of mice, including the controls (P<0.05). By contrast, the concentrations of Th2 cytokines (L-4 and IL-5) in sera of chronic infection group were significantly higher than those in the control group (P<0.05). The acute infection group did not show significant differences in concentrations of cytokines with the control group. After treatment with PZQ, the concentrations of Th1 cytokines slowly rose in the treated groups. At 12 w after treatment, the concentrations of Th1 cytokines reached the levels of control group (P>0.05). The concentration of Th2 cytokines slowly declined after treatment with PZQ, and returned to the levels of control group at 8 weeks (IL-4) or 12 weeks (IL-5) after treatment (P>0.05).

### Th1/Th2 Cytokine Profile in Spleen Cell Culture Supernatant

The concentration of cytokines in spleen cell culture supernatants unstimulated or stimulated with ConA or HBsAg were measured. The results show that mice in the chronic infection group produced significantly lower HBsAg-induced Th1 cytokines (IFN-γ and IL-2) and Th2 cytokines (IL-4 and IL-5) compared with controls (P<0.05). Deworming lead to slowly increasing levels of Th1 and Th2 cytokines, and the levels of the cytokines reached the levels of control group at 16 weeks after the treatment (P>0.05) (Fig. 3) The results indicate that a chronic *S. japonicum* infection inhibits the production of HBsAg induced Th1 and Th2 cytokines.

### Th1/Th2 Cytokine mRNA Expressing Profile

As shown in Fig. 4, the levels of IFN-γ and IL-2 mRNA in chronic group were significantly lower than those in the control group (P<0.05), which suggests that a decreased Th1 cellular immunity response occurred during chronic schistosome infection. On the other side, the levels of IL-4 and IL-5 mRNA in the chronic infection group were significantly higher than those in the control group (P<0.05). There were no significant differences among mRNA expression for all Th1/Th2 cytokines between the acute and control groups. On the other hand, the ratios of IFN-γ and IL-2 mRNA in the chronically infected mice increased after PZQ treatment while IL-4 and IL-5 mRNA ratios declined. The ratios of IFN-γ mRNA, IL-2 mRNA, IL-4 mRNA and IL-5 mRNA returned to the levels of control groups (P>0.05) at 4 (IFN-γ and IL-4 mRNA) or 8 weeks (IL-2 and IL-5 mRNA) after deworming. The expression of Th1/Th2 cytokines mRNA in each group confirmed the cytokine profile in sera and spleen cell culture supernatants.

## Discussion

Vaccination is an important measure to control infectious diseases but absent or weak responses to vaccines represent a problem. It has been proved that chronic helminth infection is one of contributing causes for absent or weak response to some vaccines [9], [10]. Also the effects of schistosomal infection on HBs vaccination were reported. Ghaffar [23] found that schoolchildren with positive stools for *S. mansoni* eggs, and age- and sex-matched children with negative stools in Egypt were given 3 doses of plasma derived hepatitis B vaccine, at 9 months after vaccination both the number of responders and the mean anti-HBs antibody titres were significantly higher in the control group than those in the group infected with *S. mansoni*,and similar results were found in adults with schistosomiasis japonica in China [21]. In addition, Ghaffar [24] reported that the immune response to hepatitis B vaccination of neonates born to mothers infected with schistosomiasis was impaired, because infections with intracellular pathogens are primarily controlled by Th1 type immune responses. For successful vaccination against most bacterial and viral diseases, an efficient Th1 response is required [25]. However, chronic helminth infections are characterized by skewing towards a Th2 type response [26], [27]. Previous studies reported that this immune modulation from Th1 to Th2 induced by chronic helminth infection impaired the specific immunity induced by Tetanus toxoid [28], Salmonella typhi [29], BCG [26] and HIV vaccines [30].

Normally HBV vaccination induces high frequencies of HBsAg-specific B and Th1 cells. These responses are associated with high serum anti-HBs antibodies levels of the subclasses immuno-globulin G1 (IgG1) and IgG2 that are driven by Th1 cytokines IL-2 and IFN- [31], [32]. A failure to produce anti-HBs antibodies is associated with an insufficient HBs antigen–specific Th1 cell response [31]. In the present studies, we demonstrated that chronic *S. japonicum* infections were characterized by skewing towards a Th2 response and the levels of Th1 cytokines were obviously lower than those of normal mice and acutely infected mice. Subsequently, the mice with chronic *S. japonicum* infection failed to generate high levels of antibodies after vaccination compared to the normal mice and acute infective mice. The results suggest that a chronic *S. japonicum* infection inhibited the immune response to the vaccine against hepatitis B in mice.

It has been demonstrated that deworming could enhance the immune response to vaccines [12], [30], [33]. In the present study, we demonstrate that, after treatment with PZQ, the quantity of anti-HBs–antibodies in sera of mice with chronic *S. japonicum* infection gradually increases and the Th2-biased profile slowly tapered. The levels of anti-HBs–antibodies and the concentration of Th1 and Th2 cytokines reached normal levels until 16 weeks after deworming. As for the possible reason of the delayed recovery of the anti-HBs immune responses after the deworming reatment with PZQ, it may be related to a switch between Th1 and Th2 responses because chronic helminth infections induce predominantly Th2-type immune responses [34]. However, protective immunity against intracellular pathogens requires Th1 responses [35]. As Th1 and Th2 responses are mutually exclusive, helminths by favouring a Th2 response may cross-inhibit a Th1 response [34]. Borkow et al. showed that the period of recovery of DTH-positive responses to PPD in humans with helminth infection was 6 months after deworming [36]. Borkow et al. also reported that eosinophilia, blood IgE, immune-activation as well as blood T-cell subsets of Ethiopian immigrants to Israel trended to normalize 6–12 months after deworming [37].

Co-infection of schistosome and HBV is a frequent event in developing countries [38]. Deworming is helpful to improve the effect of vaccination. PZQ is effective, economic and does not cause relevant side effects for treatment of schistosome infection [39]. The current studies in mice suggest that evaluation of the effects of PZQ treatment on HBV vaccine responses should now be undertaken in the human population.
